# Epidemiology of Ocular Malignancies Among the Lebanese Population: A 12-Year Review

**DOI:** 10.7759/cureus.21593

**Published:** 2022-01-25

**Authors:** Dany Akiki, Said El Hage, Jad El Masri, Wassef Chanbour

**Affiliations:** 1 General Medicine, Faculty of Medical Sciences, Lebanese University, Beirut, LBN; 2 General Medicine, Faculty of Medical Sciences, Lebanese University, Hadath, LBN; 3 Neuroscience, Neuroscience Research Center, Faculty of Medical Sciences, Lebanese University, Beirut, LBN; 4 Ophthalmology, Beth Israel Deaconess Medical Center, Boston, USA

**Keywords:** lebanon, epidemiology, incidence rate, eye, cancer

## Abstract

Purpose

Our article aims to assess the epidemiology of eye cancer in Lebanon and compare it with other regions worldwide and to study its future trends among Lebanese males and females.

Methods

Data on eye cancer cases from 2005 to 2016 were obtained from the Lebanese National Cancer Registry (NCR). Age-specific rates, crude rates, and age-standardized rates (ASRs) were subsequently calculated. Joinpoint was used to determine the changes in the slope of trends. A projection for the next 14 years was predicted using linear and logarithmic regression models.

Results

Among all tumors, eye cancer ranked 40 in females and 41 in males. The eye cancer ASR was 0.24 and 0.22 per 100,000 in males and females, respectively. However, the mean age of eye cancer was 31.94 years in males and 22.04 years in females. The cumulative risk between 0 and 74 years was 0.02%. From 2004 till 2016, a negative trend of eye cancer was observed, with a parallelism of trends between males and females. Age-specific rates showed a bimodal distribution in males. The first cluster was witnessed in the age group of 0-4 years and the second one in those above 50. An additional cluster of distribution was observed in females between 35 and 44 years of age. Forecasts for the next 14 years revealed a steady rate of eye cancer incidence of about 0.2 per 100,000. Moreover, Lebanon showed a relatively low eye cancer ASR compared to other regions worldwide, especially Zimbabwe with 5.8 and 4.8 per 100,000 in females and males, respectively.

Conclusion

Ocular malignancies showed a negative trend of incidence. A 14-year projection predicts a steady incidence rate in Lebanon and worldwide. Eye cancer seems to be affected by many risk factors. Future efforts are needed for a better understanding of the disease and a better outcome.

## Introduction

Ocular malignancies are rare. Till now, little is known about the distribution and the determining factors of these diseases [1]. In 2014, a large study, the Cancer Incidence in Five Continents, showed a worldwide distribution of the annual incidence ranging from 0.1/100,000 to 7.4/100,000 persons [2]. The incidence rates in the west are generally higher than those described in Asia [3]. Therefore, the annual incidence rate and prevalence of ocular cancer in the U.S are about 1/100,000 and 12/100,000, respectively [1]. However, in Singapore, Taiwan, Shanghai, and Japan, the annual age-standardized incidence is around 1.8 to 3 persons per million population [3,4].

Different types of cancer affect the eye, including uveal and cutaneous melanoma, squamous cell carcinoma, lymphoma, and retinoblastoma [5]. The two most common ones are retinoblastoma in children, accounting for 2% of all childhood cancer, and uveal melanoma in the elderly [6]. Besides, numerous factors seem to raise the patient’s risk of developing these cancers, such as age, race, and sunlight. For example, intraocular melanoma is more frequent in white compared to black. Also, some genetic mutations may predispose the patient to cancers, such as ocular melanoma in *BAP1* gene mutation [7]. As a result, uveal melanoma is approximately seven times more common than retinoblastoma in the United States and three to four times less common in India, Africa, South America, and Asia [8].

Nowadays, even with varying levels of the requested information, Lebanese patients are more interested in understanding their cancer status [9]. However, only two studies showed similar epidemiology and genetics of retinoblastoma at the American University of Beirut compared to the western world [10]. Thus, a detailed epidemiology of ocular cancer in Lebanon has not yet been reported. This study aims to establish the epidemiology of ocular cancer in Lebanon from 2005 to 2016 and assume its future trends among males and females. Also, it will compare the incidence of ocular cancer with regional and worldwide countries, trying to figure out the possible underlying risk factors.

## Materials and methods

Data and ethical consideration

Cancer data were retrieved from the Lebanese National Cancer Registry (NCR). Data encompassed ocular cancer cases from 2005 to 2016 and were categorized into five-year age groups and gender. The NCR tables are publicly available on the Lebanese Ministry of Public Health (MOPH) website [11]. Hence, an Institutional Review Board (IRB) approval was not necessary.

Incidence rates calculations

Data regarding the population size of Lebanon were collected from the latest World Population Prospect [12] and included the population size of each five-year age group and gender. Age-specific rates were subsequently calculated by dividing the number of ocular cancer cases in a certain age group by its respective population size, in a certain period, and gender. Therefore, age-specific rates represent the number of new cases per age group. Accordingly, the crude rate was calculated by dividing the total number of cases in a certain period by its corresponding population size. All rates were expressed as the number of cases per 100,000 persons.

Standardization procedure

To be able to compare our data with other countries, we used a standardization procedure consisting of a “standard” age composition for all countries for incidence rates. The study “World Standard Population” in 1950 was used as a reference population [13]. Age-specific rates that have been standardized, were then summed together to obtain the age-standardized rate (ASR) in a certain period. The ASR was subsequently corrected for cases of unknown age by multiplying the ASR based on cases of known age group by T/K, where T is the total number of cancer cases and K is the number of cancer cases of known age. A detailed report on the process of standardization can be found in the Cancer Incidence in Five Continents manual [2].

Cumulative rate and risk calculation

The cumulative rate, expressed in %, is the sum of age-specific rates till a certain age value. In our data, the cumulative rate is calculated by the sum of age-specific rates between 0 and 74 multiplied by 5, taking into consideration that each age group is composed of five years. The cumulative risk of cancer is the probability of being diagnosed with a certain type of cancer during a person’s lifetime. In fact, the cumulative risk is approximated by the cumulative rate when the latter is less than 10%, which is the case in our study. The standard error of the cumulative risk/rate is calculated by the following formula: $500\sqrt{di/yi^{2}}$, where *di* is the number of cases in a certain age group and *yi* is the respective population size of this age group during the studied period.

Trend analysis

The age-specific rates, crude rates, and ASRs were analyzed using the Joinpoint Regression software provided by the National Cancer Institute (NCI) and commonly used in analyzing cancer trends [14]. The regression was used to describe the trends of ocular cancer through the studied period according to all the population and genders separately. Average annual percent changes (APCs) of the different rates were gathered with their respective p-values. A significance level of 0.05 was used as a cut-off. An average APC above 0.5% is considered as having a rising trend and an APC less than -0.5% is considered as having a declining trend.

Moreover, an advanced trend analysis using a piecewise comparative approach was used for studying gender dynamics. This analysis consisted of two tests: the test of parallelism, which compares the slopes of the regression means functions between males and females, and the test of coincidence, which studies whether the overall incidence between males and females is similar. A significant test of parallelism/coincidence (p < 0.05) rejects the assumption [15].

Cancer forecasts

Linear and logarithmic regression models are the most concrete methods for cancer incidence forecasts [16]. Unlike models based on assumptions of a normal distribution, the logarithmic model assumes a Poisson distribution of the number of cases, resulting in a more reliable prediction [17].

Incidence rates of ocular cancer in Lebanon were estimated for the 2017-2030 period using the calculated incidence rates available from 2005 to 2016. The crude rates of each year, which represent the number of ocular cancer in Lebanon per 100,000, were plotted on the vertical axis with the respective years in the horizontal axis. This logarithmic model was applied using Excel to predict the incidence rates for the 14-year timeframe for males, females, and the total Lebanese population.

Comparison

To put the results of this study in context, a comparison with regional and worldwide countries was needed. Therefore, the Cancer Incidence in Five Continents Volume XI (CI5XI) database, a joint effort between the International Agency for Research on Cancer (IARC) and the International Association of Cancer Registries (IACR), provided us with high-quality statistics on the incidence of cancer in many countries [2]. Retrieved data included age-specific rates, ASRs, and crudes rates of the different countries for both males and females.

## Results

Ocular cancer ranks

The most common cancers in Lebanon in terms of cases are prostate, lung, and bladder cancer in males, and breast, lung, and colon cancer in females. However, ocular cancer is infrequent, with a mean incidence of 11.54 cases per year. During the 11 years, ocular cancer accounts for 65 cases (rank 41) and 62 cases (rank 40) in males and females, respectively (Table 1). In fact, this cancer is ranked among other infrequent cancers such as lip, adrenal gland, and anal cancer.

**Table 1:** Ranks of cancer in Lebanon by the number of cases between 2005 and 2016

Ranks	Male		Female	
	Cancer	Cases	Cancer	Cases
1	Prostate	9790	Breast	24080
2	Trachea bronchus and lung	7934	Trachea bronchus and lung	3636
3	Bladder	7256	Colon	3608
…				
30	Tongue	220	Small intestine	200
31	Small intestine	211	Leukemia unsp.	166
32	Salivary glands	184	Tongue	154
33	Mouth	174	Other female genital	143
34	Mesothelioma	167	Mouth	141
35	Nose, sinuses, etc.	105	Esophagus	141
36	Renal pelvis	93	Vagina	116
37	Penis	84	Salivary glands	112
38	Other urinary organs	72	Nasopharynx	112
39	Anus	70	Adrenal gland	63
40	Kaposi sarcoma	67	Eye	62
41	Eye	65	Nose, sinuses, etc.	60
42	Lip	61	Anus	59
43	Adrenal gland	55	Renal pelvis	41
44	Ureter	54	Mesothelioma	37
45	Tonsil	47	Lip	25

Mean age, cumulative risk, age-specific rates, and crude rates

Average ASRs of ocular cancer are 0.24 and 0.22 per 100,000 in males and females, respectively. The mean age of ocular cancer is higher in males than in females (31.94 years vs 22.04 years). In addition, the cumulative risk of ocular cancer between 0 and 74 years was estimated to be 0.02% (0.0032) (Table 2).

**Table 2:** APC of ocular malignancies between 2005 and 2016 in Lebanon, with parallelism and coincidence tests Significant difference at α = 0.05 level. *A p-value of ≤0.05 on the parallelism test indicates that the trends (slopes) between males and females are significantly different. †A p-value of ≤0.05 on the coincidence test indicates that the difference in the rates (position of trend lines) between males and females are significantly different. APC, annual percentage change; ASR, age-standardized rate

	Male	Female	Both
Total ASR	0.24	0.22	0.23
APC (%)	5	-3.4	-0.8
CI	-3.5 to 14.3	-14.4 to 9.1	-9.3 to 8.5
p-value	0.2	0.5	0.8
Trend	Rising	Declining	Declining
Parallelism* (p-value)	0.287		-
Coincidence† (p-value)	<0.001		-
Cumulative risk (%)	0.02	0.01	0.02
Standard error	0.0019	0.0022	0.0032
Mean age (years)	31.94	22.05	27.11

**Figure 1:**
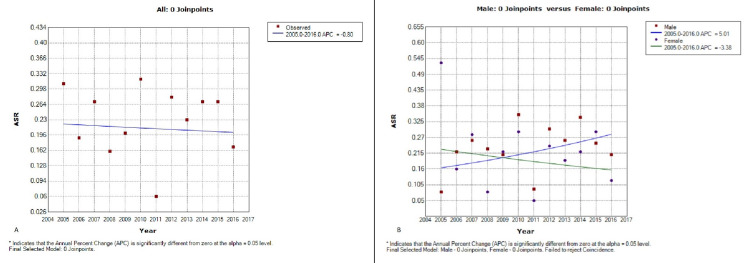
Trends of ocular malignancies in Lebanon between 2005 and 2016. (A) Both. (B) Male and female.

Moreover, ocular cancer showed a positive trend in males and a negative trend in females for the period between 2005 and 2016. Overall, a negative trend of ocular cancer is observed in both males and females. Meanwhile, there is evidence of parallelism of the trends between males and females (p = 0.287) and no coincidence of the trends between males and females (p < 0.001). (Figure 1 and Table 2).

Age-specific rates reveal a bimodal distribution of ocular cancer. The first cluster of ocular cancer was witnessed in children, particularly in the age group of 0-4 years (age-specific rate = 0.67 per 100,000). The second cluster of ocular cancer was found in individuals above 50 years of age, reaching the highest rate in the age group of 70-74 years (age-specific rate = 0.71 per 100,000). Age-specific rates of males were slightly higher than females (Table 3). However, besides the bimodal age distribution of ocular cancer, females had an additional concentration of cases between 35 and 44 years of age (35-39 age-specific rate= 0.13 per 100,000, 40-44 age-specific rate = 0.05 per 100,000) (Table 3). Lastly, crude rates (0.20 per 100,000) meaning rates before standardization were similar to age-specific rates (0.23 per 100,000) (Table 3).

**Table 3:** Age-adjusted rates and age-standardized rates for all, male, and female ocular malignancies cases according to age groups and per year in Lebanon between 2005 and 2016 y, years

Gender	Year	0-4 y	5-9 y	10-14 y	15-19 y	20-24 y	25-29 y	30-34 y	35-39 y	40-44 y	45-49 y	50-54 y	55-59 y	60-64 y	65-69 y	70-74 y	75+ y	Crude rate	ASR
Both	2005	0.94	0.41	0.19	0.44	0.00	0.00	0.00	0.00	0.00	0.00	0.00	0.00	0.00	0.94	2.27	0.00	0.28	0.31
	2006	0.48	0.00	0.19	0.00	0.00	0.00	0.00	0.00	0.00	0.40	0.95	0.64	0.00	0.00	0.00	1.09	0.17	0.19
	2007	1.53	0.00	0.20	0.00	0.23	0.00	0.00	0.00	0.00	0.00	0.93	0.00	0.00	0.00	0.00	0.00	0.21	0.27
	2008	0.54	0.45	0.00	0.00	0.00	0.00	0.00	0.28	0.00	0.00	0.00	0.00	0.00	1.05	0.00	0.00	0.13	0.16
	2009	0.28	0.47	0.00	0.00	0.00	0.00	0.00	0.00	0.00	0.00	0.00	0.00	0.00	0.00	2.14	1.75	0.19	0.20
	2010	1.62	0.47	0.00	0.00	0.00	0.00	0.00	0.00	0.00	0.00	0.00	0.00	0.71	1.04	1.06	0.00	0.22	0.32
	2011	0.00	0.23	0.00	0.00	0.00	0.00	0.00	0.27	0.00	0.00	0.00	0.00	0.65	0.00	0.00	0.00	0.06	0.06
	2012	1.31	0.00	0.00	0.00	0.00	0.22	0.00	0.00	0.26	0.00	0.00	0.45	0.00	0.00	0.00	1.41	0.23	0.28
	2013	0.38	0.39	0.37	0.00	0.00	0.00	0.00	0.00	0.00	0.00	0.00	0.00	0.00	0.81	1.08	0.00	0.19	0.23
	2014	0.52	0.00	0.00	0.00	0.00	0.00	0.00	0.45	0.25	0.00	0.31	0.40	0.00	1.48	1.06	1.85	0.26	0.27
	2015	0.82	0.69	0.17	0.33	0.00	0.00	0.00	0.00	0.00	0.00	0.29	0.38	0.00	0.68	0.00	0.59	0.24	0.27
	2016	0.00	0.17	0.00	0.16	0.16	0.00	0.00	0.00	0.00	0.00	0.28	0.36	0.45	0.00	0.98	0.57	0.16	0.17
	Total	0.67	0.27	0.09	0.08	0.03	0.02	0.00	0.09	0.05	0.03	0.22	0.20	0.16	0.51	0.71	0.64	0.20	0.23
Male		0.70	0.13	0.09	0.00	0.04	0.04	0.00	0.04	0.04	0.04	0.17	0.24	0.33	0.87	0.92	0.86	0.20	0.24
Female		0.63	0.42	0.09	0.15	0.03	0.00	0.00	0.13	0.05	0.00	0.29	0.16	0.00	0.15	0.51	0.47	0.19	0.22

Forecasts

**Figure 2:**
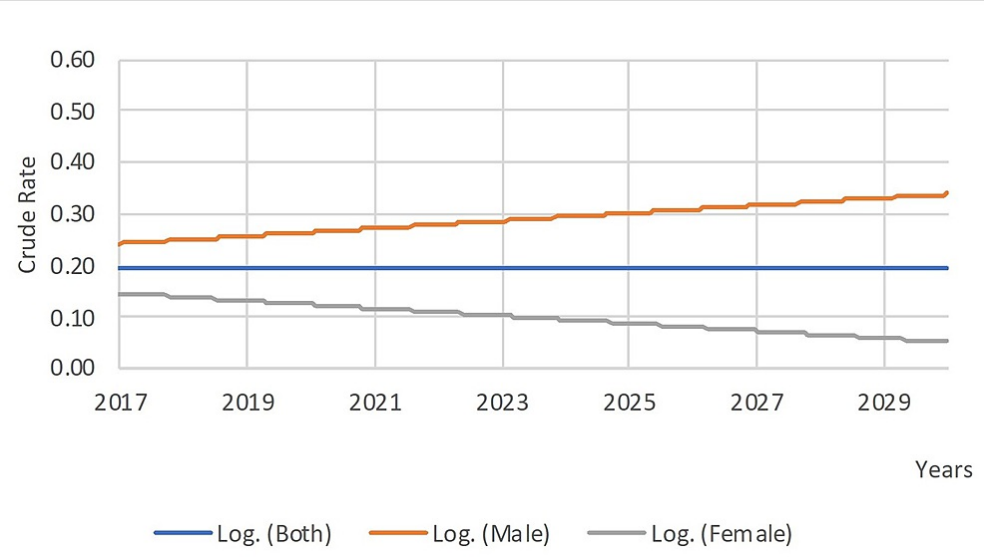
A 14-year ocular malignancies incidence projection in Lebanon

Logarithmic forecasts of ocular cancers incidence from 2017 till 2030 are shown in Figure 2. Crude rates of ocular cancer seemed to be steady at a rate of 0.2 per 100,000 (R2 =0.0001). However, a closer look at gender forecasts revealed a slight increase of rates in males from approximatively 0.24 per 100,000 in 2017 to 0.33 per 100 000 in 2030 (R2 =0.192) and a slight decrease in females from approximatively 0.15 per 100,000 in 2017 to 0.06 per 100,000 in 2030.

Comparison

Comparison of age-specific rates, crude rates, and ASR of ocular cancer in females and males in Lebanon with other countries are presented in Tables 4, 5, respectively.

**Table 4:** Comparison of females in terms of age-adjusted rates and age-standardized Rates of cancer in Lebanon with regional and random countries y, years

	Countries	0-4 y	5-9 y	10-14 y	15-19 y	20-24 y	25-29 y	30-34 y	35-39 y	40-44 y	45-49 y	50-54 y	55-59 y	60-64 y	65-69 y	70-74 y	75+ y	80-85 y	85+ y	Crude rate	ASR
Regional countries	Lebanon	0.63	0.42	0.09	0.15	0.03	-	-	0.13	0.05	0.00	0.29	0.16	0.00	0.15	0.51	0.47			0.19	0.22
	Algeria (Setif)	1.5	-	-	-	-	-	-	-	-	0.7	-	-	-	-	-	-			0.2	0.2
	Bahrain	0.6	-	-	-	-	-	-	-	-	-	-	-	3.5	-	-	-			0.1	0.2
	Jordan	1.1	0.2	-	-	0.2	-	0.1	0.4	0.1	0.6	-	0.6	1.1	-	1.4	-			0.3	0.4
	Kuwait	0.1	-	-	-	-	-	-	-	-	-	-	0.6	-	-	-	-			0	0
	Qatar	-	-	-	-	-	-	-	-	-	-	-	-	-	-	-	-			0	0
	Saudi Arabia (Riyadh)	2.9	0.1	0.2	-	-	-	-	0.1	-	-	0.2	0.4	0.6	-	2.1	-			0.4	0.5
Random countries	Japan (Aichi Prefecture)	1.2	0.1	-	-	0.1	-	-	0.1	-	0.1	0.1	-	0.1	0.2	0.8	-			0.2	0.2
	Philippines (Manila)	1	0.1	0.1	-	-	-	-	-	-	-	-	0.2	0.3	-	1.1	-			0.2	0.2
	France (Bas-Rhin) (2008-2011)	1.5	-	-	-	-	-	-	-	1.2	2.5	1.9	-	1.7	2.2	4.6	1.2	4.4	4.7	1.1	0.8
	Germany (Bavaria)	0.8	0.1	-	-	-	-	0.1	0.3	0.4	0.6	1.2	0.8	1.1	1.5	2	2	3.4	2.1	0.8	0.5
	Ukraine	0.9	0.2	0.1	-	0.1	0.1	0.3	0.3	0.5	0.8	1	1.1	1.7	1.4	1.4	1.4	1.2	0.9	0.7	0.5
	Slovenia	0.8	-	-	-	0.3	0.3	-	0.3	0.3	1	0.5	2.4	3.7	0.4	2.4	1.8	-		1	0.6
	Zimbabwe	2	-	-	-	0.3	2.9	7.4	6.3	17.7	15.5	25.5	7.7	7.6	11.1	-	-			6.3	5.8
	Canada (Alberta)	2	0.2	-	-	0.1	0.4	-	0.2	0.8	0.4	1.6	1.1	2.3	1.6	2.1	4.4	3.1	3.6	0.9	0.8
	USA (national)	1.4	0.1	-	0.1	0.1	0.1	0.2	0.3	0.4	0.6	1	1.1	1.5	2	2.2	2.5	2.4	2.3	0.8	0.6
	Argentina (Chaco)	0.4	0.4	-	-	-	-	-	-	-	0.7	2.3	0.9	-	1.5	1.7	2.3	-		0.4	0.4
	Jamaica (2008-2011)	2.3	-	-	0.8	-	-	-	-	-	0.9	-	-	2.4	-	-	4.9	-	-	0.4	0.5

**Table 5:** Comparison of males in terms of age-adjusted rates and age-standardized rates of cancer in Lebanon with regional and random countries. y, years

	Countries	0-4 y	5-9 y	10-14 y	15-19 y	20-24 y	25-29 y	30-34 y	35-39 y	40-44 y	45-49 y	50-54 y	55-59 y	60-64 y	65-69 y	70-74 y	75+ y	80-85 y	85+ y	Crude rate	ASR
Regional countries	Lebanon	0.70	0.13	0.09	-	0.04	0.04	0.00	0.04	0.04	0.04	0.17	0.24	0.33	0.87	0.92	0.86			0.20	0.24
	Algeria (Setif)	0.7	-	-	-	-	-	-	-	0.6	-	0.8	-	-	2.4	2.6	-			0.2	0.3
	Bahrain		0.7	-	-	-	-	-	-	-	-	-	-	-	-	-	-			0.1	0.1
	Jordan	1.2	0.1	-	-	0.1	0.1	-	0.1	0.8	0.8	0.3	-	1.3	0.9	0.7	-			0.3	0.4
	Kuwait	0.5	-	-	-	-	-	0.1	-	-	0.1	-	-	0.6	-	-	-			0.1	0.1
	Qatar		-	-	-	-	-	-	-	-	4	-	-	-	-	-	-			0.2	0.2
	Saudi Arabia (Riyadh)	2.4	-	0.3	0.1	-	-	-	0.4	-	0.2	-	-	0.9	0.7	-	-			0.4	0.5
Random countries	Japan (Aichi Prefecture)	2	-	-	-	-	-	0.1	0.1	0.1	-	-	0.2	0.4	0.3	0.2	0.6	0.5	0.9	0.2	0.3
	Philippines (Manila)	1.4	0.3	0.1	-	0.1	0.1	0.1	-	-	0.1	-	0.6	0.3	0.5	2.7	-			0.3	0.4
	France (Bas-Rhin) (2008-2011)	2.2	-	-	-	-	0.7	-	-	-	1.9	3.3	1.4	0.8	3.6	4.1	5.1	2.7	-	1.2	0.9
	Germany (Bavaria)	0.7	0.1	-	-	0.1	0.3	0.1	0.2	0.2	0.8	0.8	1.8	2.4	2.6	3.4	3.2	4.9	4.9	1.1	0.6
	Ukraine	1.1	0.1	-	0.1	0.1	0.1	0.3	0.3	0.4	0.6	1.1	1.4	2	1.9	1.9	1.9	1.5	1.5	0.7	0.6
	Slovenia	2.6	-	-	-	0.3	-	-	0.5	-	2	1	1.3	1.7	4	2.1	2.2	-		1.1	0.9
	Zimbabwe	2.4	0.5	-	-	0.9	2.7	5.2	7.3	1	13.1	15.9	15	3.8	6.1	8.4	-			3.6	4.8
	Canada (Alberta)	1.6	-	-	-	-	-	0.1	0.4	0.7	0.7	1.4	2.1	1.8	2.6	5.9	9.8	7.6	3.7	1.1	0.9
	USA (national)	1.3	0.2	-	-	0.1	0.1	0.2	0.3	0.5	0.7	1.1	1.6	2.1	3	3.4	4	4.2	4.1	0.9	0.7
	Argentina (Chaco)	1.1	0.4	-	-	-	-	0.5	-	-	-	0.8	0.9	-	1.7	6.8	3.4	-		0.5	0.5
	Jamaica (2008-2011)	1.1	-	-	-	-	-	1.1	-	1.1	-	-	-	-	-	-	6.1	-	-	0.3	0.3

Lebanon showed relatively a low ASR in females (ASR = 0.22 per 100,000) compared to Saudi Arabia (ASR = 0.5 per 100,000) and Jordan (ASR = 0.4 per 100,000). Likewise, when comparing to other countries such as Zimbabwe (ASR = 5.8 per 100 000), France and Canada (ASR = 0.8 per 100,000), and Slovenia and the USA (ASR = 0.6 per 100 000). Yet Lebanon showed a high ASR of ocular cancer in females when compared to Kuwait and Qatar, who registered the lowest and almost null ASR regionally and worldwide (Table 4).

Similarly, ASRs in Lebanese males (ASR = 0.24 per 100,000) were predominantly lower than other countries, notably Saudi Arabia (ASR = 0.5 per 100,000), Jordan (ASR = 0.4 per 100,000), and Algeria (ASR = 0.3 per 100,000) and also when comparing to other countries such as Zimbabwe (ASR= 4.8 per 100 000), France, Canada, and Slovenia (ASR = 0.9 per 100,000). However, Lebanon showed a high ASR of ocular cancer in males when compared to Bahrain and Kuwait (ASR = 0.1 per 100,000) (Table 5).

## Discussion

Even though ocular cancer remains one of the rarest tumors in Lebanon, ranking 40 in females and 41 in males (Table 1). It is still interesting to establish its epidemiology among the Lebanese population.

The World Cancer Research Fund (WCRF) classified Lebanon in the first 50 nations to have the highest ASR in all types of cancer, where Lebanon ranked 48 with an ASR of 242.8 per 100,000 [18]. Meanwhile, the highest cancer rate was registered in Australia 468 per 100,000, with a corresponding ASR of ocular cancer of 1.3 per 100,000. The same was for France and Germany ranking 7 and 15 with a total ASR of 344.1 and 313.1 per 100,000, respectively [18]. This will let us conclude that the ASR of ocular cancer is varying harmoniously with the ASR of all types of cancer.

Contrariwise, African nations scoring extremely high levels of ocular cancer ASR are at the same time scoring low cancer ASR, 154.2 per 100,000 in Malawi, 200.4 per 100,000 in Zimbabwe, and 153.8 per 100,000 in Uganda compared to Australia, France, and Germany [18]. Besides, a study in Zimbabwe showed that retinoblastoma is the third most common cancer of childhood [19]. This will lead us to confirm the presence of additional risk factors in the African countries affecting specifically ocular cancer and raising its incidence above the expected one. At this point, we will stress the fact that according to the Zimbabwe National Cancer Registry, 60% of cancers were associated with HIV infections, which is an extremely high percentage, including squamous cell carcinoma of the conjunctiva. A study in Uganda revealed a 10-fold increased risk of conjunctival cancer in HIV-infected individuals [20]. Then knowing the role of UV light in uveal melanoma [21], the ASR of ocular cancer would be even higher without the protective effect of skin pigmentation where the darker irides transmit less UV light having a high total concentration of melanin [22].

Lebanon showed an overall negative trend of ocular cancer, with evidence of parallelism of trends between males and females. Similarly, a previous study conducted in the USA from 1955 to 1989 presented a fall in incidence rates of ocular cancer and relatively constant incidence rates in England and Wales [23]. Also, an international study revising data from 1983 till 1997 showed a decrease in uveal melanoma from 82% to 75% [24]. In regard to retinoblastoma, the age-adjusted incidence rate of retinoblastoma in the USA has remained stable for over 30 years [25]. The same is for Canada and other nations [26]. The continuity of trends of ocular cancer among different countries confirms the fact that there is either a decrease or even a constant trend of ocular cancer, but rarely a positive trend of ocular cancer.

Age-specific rates show a bimodal distribution in males; the first cluster is witnessed in the age group of 0-4 years and the second one above the age of 50 years. An additional cluster of distribution was observed in females between 35 and 44 years of age (Table 3). This third cluster in middle-aged women explains the low mean age of ocular cancer compared to men. Bhaumik et al. demonstrated the increased risk of ocular melanoma among women who had ever been pregnant [27]. On the contrary, Holly et al. observed a decreased risk among the same categories. They both agreed that no change in the risk among women who had used oral contraceptives. Moreover, decreased risk among women who had undergone oophorectomy [27,28]. Contrary to everything declared previously, Behrens et al. did not observe any association between uveal melanoma and all of the factors already mentioned, including reproductive history, hormonal exposure, and oophorectomy [29].

Even though forecasts of ocular cancer in Lebanon for the next 14 years revealed a steady rate of incidence of about 0.2 per 100,000 (Figure 2), it is still crucial to consider the importance of an early diagnosis and routine ophthalmoscopy instead of an advanced stage of the tumor and poorer prognosis [10]. This was emphasized by a study on retinoblastoma cases in Lebanon, which showed that 10% of retinoblastoma cases in Lebanon had a past familial history of the disease [10]. Another similar study found that enucleations would be avoided if retinoblastoma is detected early in the course of the disease [11]. While concerning ocular melanoma and other primary ocular tumors, we remarkably detected a lack to absent research activity in Lebanon. More studies must be initiated for a better understanding and control of the disease.

Limitations

This study is the first to assess the epidemiology of ocular cancer in Lebanon from 2005 to 2016. However, the available data listed by the MOPH in Lebanon registered ocular cancer as one entity without mentioning the corresponding subtypes. Therefore, our study lacks an adequate analysis of the histological variants of ocular cancer. Moreover, there is absence of data after 2017.

## Conclusions

To our knowledge, ocular cancer forecasts show steady negative trends of incidence in Lebanon and worldwide. Even though ocular cancer is rare, it seems to be influenced by many factors including tumors, ASR, HIV, UV light, gender, and hormonal factors in women. Therefore, we will appreciate future studies assessing the epidemiology and risk factors of this tumor and its different subtypes. So far, this will lead us to a better understanding of the disease and a better outcome.
